# Evaluation of choroidal parameters in eyes at the first onset of acute anterior uveitis

**DOI:** 10.1186/s12886-019-1072-7

**Published:** 2019-02-28

**Authors:** Marta P. Wiącek, Anna Machalińska

**Affiliations:** First Department of Ophthalmology, Pomeranian Medical University in Szczecin, Al. Powstańców Wielkopolskich 72, 70-111 Szczecin, Poland

## Abstract

**Background:**

Little is known about choroidal involvement in anterior uveitis. The aim of our study was to evaluate changes in choroidal thickness and volume in eyes with acute anterior uveitis (AAU) using enhanced depth imaging-optical coherence tomography (EDI-OCT) at baseline and after treatment, which were compared with healthy fellow eyes.

**Methods:**

For the study, 35 individuals with unilateral acute AAU at the first onset were enrolled. Subfoveal thickness and choroidal volume were measured with EDI-OCT in nine Early Treatment of Diabetic Retinopathy Study (ETDRS) subfields before and after the completion of treatment. Moreover, axial length measurements of both eye bulbs were determined by optical biometry.

**Results:**

No statistically significant differences in choroidal thickness or choroidal volume were detected between AAU eyes at baseline and after treatment and fellow eyes. Positive correlations between the values of anterior chamber flare and absolute CT changes in both temporal and inferior ETDRS fields, as well as in superior outer ring were detected. Negative correlations between age and both choroidal thickness and choroidal volume were detected in AAU eyes at baseline and after treatment, as well as in fellow eyes.

**Conclusions:**

Evaluation of the choroid with EDI-OCT does not appear to be a reliable tool for the treatment monitoring of eyes with anterior uveitis.

## Introduction

Both the etiology and manifestation of uveitis may vary among individuals. Acute anterior uveitis (AAU) is the most common manifestation of all uveitis types according to the International Uveitis Study Group [1, 2], which defined and presented valid nomenclature. Up to 49% of all uveitis cases are diagnosed as AAU [2]. Although the range of tissues involved in the inflammatory processes in AAU is limited to the anterior segment, the leading causes of AAU are systemic inflammations, with infectious or inflammatory etiology. Development of optical coherence tomography (OCT) technology also showed subclinical involvement of the posterior retina and choroid in some cases of AAU [2, 3]. Involvement of the choroid may cause changes in choroidal thickness (CT) or correlate with lower visual acuity in those patients [3, 4]. The OCT technology provided an opportunity to improve the monitoring of uveitic patients. Moreover, spectral-domain OCT (SD-OCT) with enhanced depth imaging (EDI-OCT) software enabled precise qualitative and quantitative analysis of the choroid.

To our knowledge, little is known about the relationship between choroidal parameters and acute AAU. Additionally, there is no agreement among researchers on the influence of AAU on CT. Available reports are based mainly on a single measurement of CT in the subfoveal region that is performed manually by the authors or, rarely, on thickness-mapping of the choroid [4]. This methodology does not cover all choroid parameters and can lead to biased results. An analysis of volume based on central 6 mm perifoveal B-scans of the choroid could provide more precise information on choroidal involvement in AAU. Usage of this novel technology may help to understand the nature of AAU and may be useful in the prevention of irreversible deterioration of vision in those patients.

The aim of our study was to determine the difference in CT and choroidal volume (CV) between eyes with acute AAU at the first onset and fellow eyes (FE). Changes in both parameters were also compared at baseline and after the treatment of uveitis in eyes with AAU. To our knowledge, this is the first report analyzing CV in AAU.

## Materials and methods

The study was planned as a prospective interventional study. Adults with a newly diagnosed, unilateral, first episode of AAU were recruited for the study. The exclusion criteria were any of the following: the diagnosis of any additional ocular disease, the use of ophthalmological drugs or a history of ocular surgery within 1 year of the study visit. Macular disease in AAU or FE eyes, such as macular edema, vitreomacular traction, central serous chorioretinopathy, age-related macular degeneration, and epiretinal membrane or diabetic retinopathy, was also considered an exclusion criterion. Depending on the AAU severity, a topical steroid treatment without or with antibiotics in case of a concomitant conjunctivitis (3 cases), a non-steroid anti-inflammatory or tropicamide eye drops were implemented. In the most severe cases, subconjunctival injections of steroid or epinephrine were also administered.

All individuals underwent detailed tests for associated systemic diseases. To detect potential infectious disease, the venereal disease research laboratory (VDRL) test for syphilis, *Borrelia* IgM and IgG antibodies with ELISA tests for Lyme disease, IgM and IgG antibodies for toxoplasmosis, and HIV serology test were performed. Also, complete blood count and C-reactive protein were measured. The imaging diagnostics with chest X-ray and sacroiliac joint X-ray was performed. Accordingly, the HLA tissue typing (HLA-B27) and rheumatoid factor were examined. If needed, rheumatologist examination with antinuclear antibody (ANA) and antineutrophil cytoplasmic antibody (ANCA) evaluation was performed.

The ophthalmological examination was performed at the first admittance of the patient with AAU symptoms, when AAU was diagnosed according to International Uveitis Study Group criteria [1], and after the completion of treatment. Data on age, sex and possible etiology were collected. Visual acuity (VA) was measured using a Snellen letter chart, and transformed to LogMAR (Logarithm of the minimum angle of resolution) for statistical analyses. Intraocular pressure was measured by a Pascal dynamic contour tonometer. Refractive error was measured using an autorefractometer (RC-800, Tomey, Phoenix, USA). Slit lamp examination and indirect ophthalmoscopy with a Volk lens were performed on the anterior and posterior segments at both time points. Optical biometry (IOL Master, Zeiss, Germany) was performed in order to measure the axial length of AAU eyes and FE. The retrospective analysis of the degree of the anterior chamber inflammation based on the medical record at the first admittance of the patient with AAU symptoms was performed. The anterior chamber flare was expressed in The Standardization of Uveitis Nomenclature (SUN) Working Group slit lamp grading scheme [1]. In selected cases, additional photograph of the anterior segment of the AAU eye was performed (magnification 16x, slit lamp and Topcon IMAGEnet i-base, Topcon, Tokyo, Japan). The absolute CT change was defined as difference between CT values before and after the treatment. The relative CT change was defined as difference between CT measurements in AAU eyes before the treatment and FE. Similarly, the CV absolute and relative changes were defined respectively. Both values, the absolute and relative CT changes, were correlated with the degree of intraocular inflammation.

**Fig. 1:**
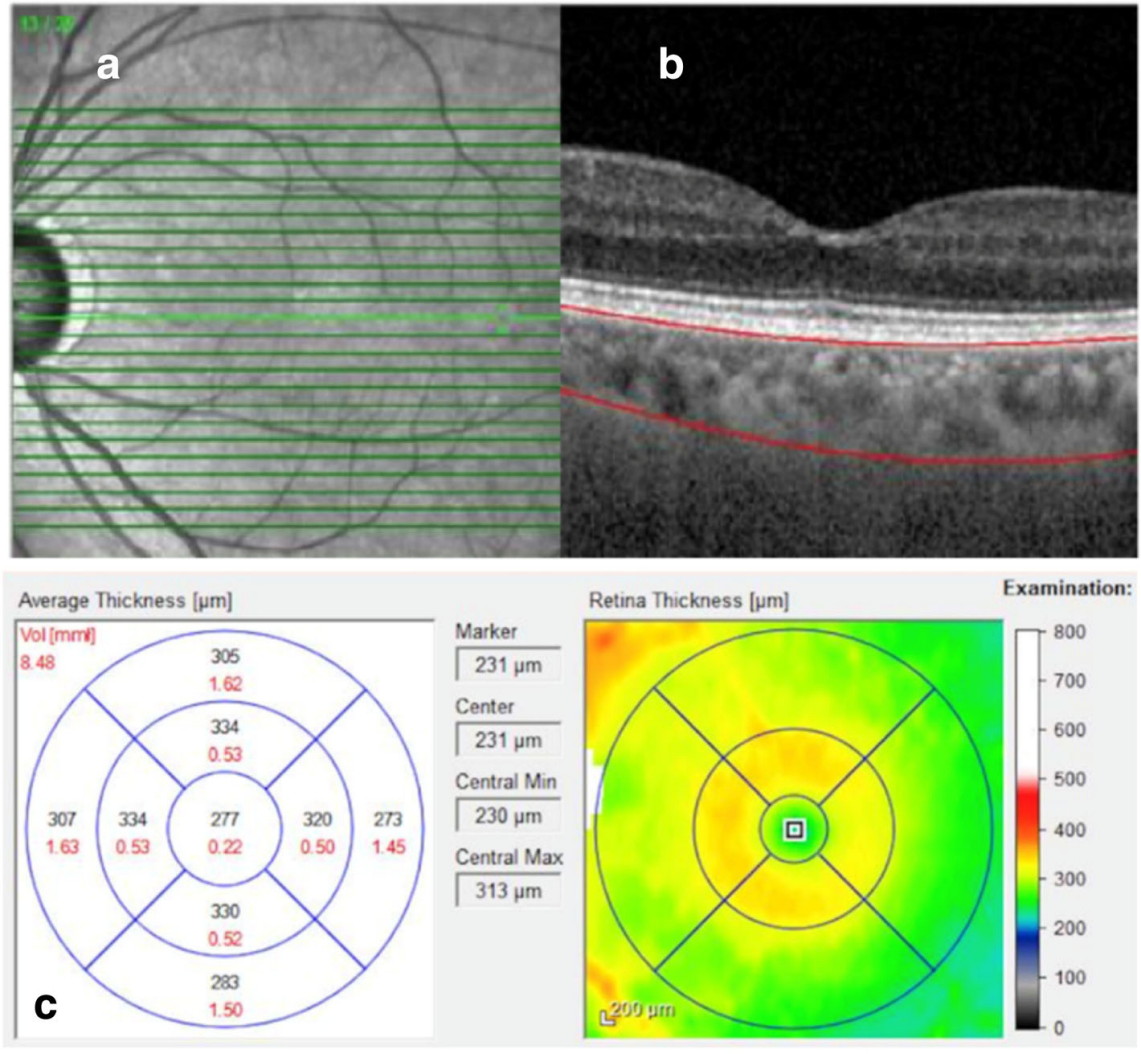
Enhanced depth imaging-optical coherence tomography (EDI-OCT) of the macular region. **a** 31 B-scans of the macular region; **b** Marked scleral-uveal junction; **c** The choroidal volume [mm^3^] (marked in red) and thickness [μm] (marked in black) measurements presented separately for 9 Early Treatment Diabetic Retinopathy Study (ETDRS) subfields

The EDI-OCT images of a macular region were obtained (Heidelberg Engineering, Heidelberg, Germany) in all subjects at the same time in the morning (time interval of 2 h). All measurements were performed by one researcher who was blinded to which eye was affected. The scleral-uveal junction was marked manually on 31 available B-scans of each examination (Fig. 1). Both CT and CV measurements were presented as the average of all points within the 9 Early Treatment Diabetic Retinopathy Study (ETDRS) subfields provided automatically by Heidelberg Engineering software. Subfoveal CT was calculated automatically as intersection of 6 radial scans centered at the foveola. The EDI-OCT examination had to reach a satisfactory level of quality with the scleral-uveal junction clearly visible.

This study was performed in accordance with the Declaration of Helsinki. Written informed consent was obtained from all individuals.

### Statistical analysis

Statistical analysis was performed using Statistica 13.1 (Statsoft, Tulsa, OK, USA) software. Quantitative variables were tested for normality by Shapiro-Wilk test and by Levene’s test for equality of variances. VA in AAU eyes at baseline and after the treatment was tested using a dependent t-test for paired samples. In the case of the remaining nonparametric variables, the Wilcoxon signed-rank test was used. The Wilcoxon signed-rank test was performed to compare CT and CV in all ETDRS fields between AAU eyes, both before and after treatment, and healthy FEs. The relationship between CV or CT and the axial length of the analyzed eye bulbs was tested using simple linear regression. The correlation between CV or CT and age was tested using a Spearman’s rank correlation. Any *p* value below 0.05 was considered statistically significant. The results were presented as the mean ± standard deviation.

## Results

In this study, 35 eyes with a diagnosis of AAU from 35 individuals were examined and analyzed (21 women and 14 men). The mean age of individuals was 47.23 ± 16.53 years (ranging from 18 to 84 years). In the analyzed group, 4 cases of ankylosing spondylitis, 2 cases of juvenile idiopathic arthritis and 1 case of psoriasis were diagnosed. In 28 cases, the etiology of AAU was idiopathic. Clinical characteristics in the analyzed group are presented in Table 1. The total time of AAU treatment and the interval between OCT examinations were in the range of 1 to 6 weeks*.* At the time of the follow-up the anterior chamber flare evaluated in SUN Working Group slit lamp grading scheme was graded as no flare present (stage 0). After a course of AAU treatment, VA in the affected eye improved significantly from 0.19 ± 0.23 to 0.09 ± 0.18 (*p* = 0.0006). No significant changes in VA of the FE or in the intraocular pressure in both eyes were detected at baseline and after the AAU treatment.

**Table 1:** Clinical characteristics of the eye with acute anterior uveitis and in healthy fellow eye before and after the treatment

	Acute anterior uveitis eye			Fellow eye		
	Before	After	*p* value*	Before	After	*p* value**
visual acuity [logMAR]	0.19 ± 0.23	0.09 ± 0.18	0.0006	0.08 ± 0.17	0.08 ± 0.18	0.655
intraocular pressure [mmHg]	14.62 ± 3.64	16.36 ± 3.25	0.674	16.9 ± 2.18	16.9 ± 1.42	0.345
refraction [D]	−3.50 to + 7.00			−5.25 to + 6.00		0.726
optical biometry	23.29 ± 1.05			23.32 ± 0.99		0.911

*acute anterior uveitis eye before vs after the treatment

**acute anterior uveitis eye before the treatment vs fellow eye

Table 2 provides the values of CT and Table 3 provides the measurements of CV obtained for AAU eyes at baseline and after treatment, as well as those for fellow eyes. No significant differences in both CT and CV, between AAU eyes before and after the treatment in the 9 ETDRS subfields were observed. However, the CT was significantly higher in outer temporal (mean: 331.84 vs 279.01 μm) and lower in nasal ETDRS subfield in AAU eyes compared with healthy FE (mean: 290.69 vs 318.33 μm). Similarly, the only differences in CV were observed in the outer nasal and temporal choroidal ring areas. In the nasal outer field, the choroid was significantly thicker (mean: 1.73 vs 1.46 mm^3^), while in the temporal outer field, it was thinner (mean: 1.48 vs 1.75 mm^3^) in AAU eyes before treatment than in FEs.

**Table 2:** Mean choroidal thickness [μm] in ETDRS fields in EDI-OCT imaging in eye with anterior uveitis before and after the treatment and in healthy fellow eye

ETDRS		Anterior uveitis eye			Fellow eye	
		CT before treatment	CT after treatment	*p* value*	CT	*p* value**
Central ring area		361 ± 101.64	506.89 ± 891.37	0.347	344.57 ± 95.59	0.719
Inner ring area	Temporal	363.94 ± 97.11	356.44 ± 128.49	0.103	335.33 ± 89.49	0.123
	Nasal	351.16 ± 88.43	351.81 ± 137.46	0.073	349.31 ± 93.65	0.877
	Superior	373.62 ± 92.5	376.41 ± 126.01	0.841	367.61 ± 97.74	0.349
	Inferior	346.59 ± 90.68	351.18 ± 125.94	0.681	335.31 ± 84.46	0.688
Outer ring area	Temporal	331.84 ± 83.93	328.18 ± 112.1	0.476	279.01 ± 81.39	0.0002
	Nasal	290.69 ± 77.82	299.74 ± 122.99	0.809	318.33 ± 74.27	0.007
	Superior	362.56 ± 87.25	369.04 ± 119.75	0.611	349.65 ± 101.21	0.335
	Inferior	320.69 ± 86.06	328.07 ± 118.98	0.861	312.03 ± 90.46	0.829

*Abbreviations: CT* choroidal thickness

*anterior uveitis eye before vs after the treatment

**anterior uveitis eye before the treatment vs fellow eye

**Table 3:** Mean choroidal volume [mm^3^] in ETDRS fields in EDI-OCT imaging in eye with acute anterior uveitis before and after the treatment and in healthy fellow eye

ETDRS field		Acute anterior uveitis eye			Fellow eye	
		CV before treatment	CV after treatment	*p* value*	CV of FE	*p* value**
Central ring area		0.28 ± 0.08	0.28 ± 0.1	0.361	0.28 ± 0.07	0.567
Inner ring area	Temporal	0.55 ± 0.08	0.55 ± 0.21	0.353	0.53 ± 0.12	0.439
	Nasal	0.53 ± 0.14	0.54 ± 0.22	0.107	0.56 ± 0.13	0.129
	Superior	0.57 ± 0.15	0.58 ± 0.2	0.689	0.57 ± 0.13	0.922
	Inferior	0.53 ± 0.13	0.55 ± 0.2	0.378	0.54 ± 0.12	0.456
Outer ring area	Temporal	1.73 ± 0.41	1.76 ± 0.61	0.773	1.46 ± 0.4	0.0003
	Nasal	1.48 ± 0.41	1.53 ± 0.66	0.998	1.75 ± 0.35	0.0000
	Superior	1.89 ± 0.46	1.95 ± 0.67	0.855	1.84 ± 0.56	0.758
	Inferior	1.67 ± 0.42	1.75 ± 0.63	0.689	1.71 ± 0.45	0.641

*Abbreviations: CV* choroidal volume, *FE* fellow eye

*acute anterior uveitis eye before vs after the treatment

**acute anterior uveitis eye before the treatment vs fellow eye

To characterize the other factors that may influence the obtained results, we evaluated the potential association between the quantitative analysis of choroidal parameters and several systemic risk factors and ocular conditions. We found no correlation between axial length and CT or CV in any of the ETDRS fields. The mean anterior chamber flare expressed in SUN Working Group slit lamp grading scheme before treatment was 1.69 ± 1.44 (*N* = 13). Significant positive correlations between the values of anterior chamber flare and absolute CT changes in both temporal and inferior ETDRS fields, as well as in superior outer ring were detected (Table 4). The trend for positive correlation in central ring area was also observed. Contrary, no statistically significant correlations between the values of the anterior chamber flare and relative CT changes, or absolute and relative changes of CV in any ETDRS field were detected.

**Table 4:** Correlations between the values of anterior chamber flare expressed in the Standardization of Uveitis Nomenclature (SUN) Working Group slit lamp grading scheme and absolute choroidal thickness changes [μm] in patients with anterior uveitis

ETDRS		Absolute CT change	Correlation coefficient	*p* value
Central ring area		68.15 ± 117.83	+ 0.511	0.074
Inner ring area	Temporal	64.46 ± 112.77	+ 0.675	0.011
	Nasal	53.46 ± 110.28	+ 0.126	0.681
	Superior	51.08 ± 101.22	+ 0.462	0.111
	Inferior	61.69 ± 114.79	+ 0.755	0.003
Outer ring area	Temporal	61.85 ± 110.58	+ 0.718	0.006
	Nasal	34.46 ± 100.12	+ 0.221	0.468
	Superior	46 ± 91.31	+ 0.652	0.016
	Inferior	56.61 ± 120.86	+ 0.608	0.027

*Abbreviations: CT* choroidal thickness

Remarkably, subfoveal CT values in AAU eyes at baseline and after treatment, as well as in fellow eyes, were negatively correlated with the age of the participants (R = -0.594, *p* = 0.002; R = -0.686, *p* = 0.001; R = -0.411, *p* = 0.027; respectively). Similarly, in AAU eyes before and after treatment as well as in FEs, a negative correlations between CT and age was detected in most ETDRS subfields (Table 5). Moreover, a significant negative correlations between age and CV in AAU eyes at baseline and after treatment were detected in all the 9 ETDRS subfields (Table 6). In fellow eye a significant negative correlations between age and CV were observed in most of ETDRS subfields (Table 6). Those findings confirmed that older patients exhibited thinner choroids compared with their younger counterparts.

**Table 5:** Correlation of the age and choroidal thickness [μm] in patients with anterior uveitis - results presented as correlation coefficient

ETDRS		Anterior uveitis eye				Fellow eye	
		CT before treatment	*p* value	CT after treatment	*p* value	CT	*p* value
Central ring area		−0.367	0.046	−0.689	0.000	−0.437	0.018
Inner ring area	Temporal	− 0.341	0.065	−0.678	0.0001	−0.353	0.06
	Nasal	−0.286	0.126	−0.492	0.012	−0.497	0.006
	Superior	−0.43	0.018	− 0.663	0.0003	−0.478	0.009
	Inferior	− 0.282	0.131	−0.556	0.004	− 0.304	0.109
Outer ring area	Temporal	−0.385	0.035	−0.751	0.000	−0.386	0.039
	Nasal	−0.245	0.191	−0.359	0.077	−0.508	0.005
	Superior	−0.364	0.048	−0.651	0.0004	−0.445	0.017
	Inferior	−0.264	0.158	−0.596	0.002	−0.353	0.063

*Abbreviations: CT* choroidal thickness

**Table 6:** Correlation of the age and choroidal volumes [mm^3^] in patients with anterior uveitis - results presented as correlation coefficient

ETDRS		Anterior uveitis eye				Fellow eye	
		CV before treatment	*p* value	CV after treatment	*p* value	CV	*p* value
Central ring area		−0.438	0.015	−0.625	0.001	−0.381	0.038
Inner ring area	Temporal	−0.482	0.007	−0.616	0.001	−0.412	0.024
	Nasal	−0.481	0.007	−0.569	0.003	−0.424	0.019
	Superior	−0.612	0.0003	−0.673	0.0002	−0.509	0.004
	Inferior	−0.431	0.017	−0.529	0.005	−0.245	0.191
Outer ring area	Temporal	−0.501	0.005	−0.667	0.0002	−0.418	0.021
	Nasal	−0.476	0.007	−0.526	0.006	−0.473	0.008
	Superior	−0.561	0.001	−0.627	0.001	−0.441	0.015
	Inferior	−0.393	0.031	−0.573	0.002	−0.248	0.185

*Abbreviations: CV* choroidal volume

## Discussion

It has previously been demonstrated that CT is affected over the course of AAU. In HLA-B27 positive patients, the values of subfoveal CT were higher in eyes with AAU at the first onset compared with FEs [4]. A B-scan of the macular region in those eyes also revealed dilated large choroidal vessels. Those vessels disappeared after the AAU treatment and were not present in the FEs. Simultaneously, there was no correlation between choroidal thickening and leakage in fluorescein angiography, which is considered a sign of an active inflammatory process in the choroid. This supported the theory of subclinical inflammation of the choroid being present in AAU [4]. Interestingly, the authors used an automated measurement of CT with investigator correction if needed, but the results were not provided separately for all ETDRS fields.

In another study on HLA-B27-positive patients with a diagnosis of ankylosing spondylitis, CT in AAU eyes was found to be significantly higher than in healthy FEs and healthy control eyes [5]. However, in this study, eyes with recurrent anterior uveitis were enrolled, while only the first onset of AAU may give us an unbiased result of choroidal parameter changes. Moreover, the information on the number of AAU eyes that were affected and the number of FEs was not provided. The study was based on a single measurement of subfoveal CT performed manually [5].

Similarly, Gabriel et al. [6] noted that, in the first episode of the nongranulomatous type of AAU, both Haller’s and Sattler’s layers were enlarged over the course of AAU. After the treatment, a significant reduction in CT was observed; however, this value did not differ significantly in comparison with FEs [6].

Kim et al. [7] similarly noticed increased subfoveal CT in patients with AAU of mixed etiology compared to healthy FEs or control eyes. However, the authors did not specify if that was the first onset of AAU. Moreover, patients with more than a one-year history of AAU had FEs included. The CT measurements were performed manually by using calipers in a single EDI-OCT image.

In contrast, there are reports documenting that choroidal thickness is reduced over the course of AAU. Due to vessel atrophy, ischemic changes and local fibrosis of the choroid, the diameter of blood vessels was reduced in both Haller’s and Sattler’s choriocapillaris layers, causing the diffuse and complete thinning of CT seen in Fuchs uveitis syndrome [8, 9]. Nevertheless, all analyses were based on single measurements done with a central B-scan of every ETDRS subfield, and the total CT was not evaluated in this study. Moreover, only data from patients whose disease was inactive for longer than 6 months were analyzed, but no data on disease duration or the number of uveitic episodes were provided. The CT thinning in Fuchs uveitis syndrome was also confirmed by other researchers [9]. Accordingly, CT was measured manually using calipers on a single B-scan in the center of a subfoveal region [9].

In this study, significant changes in CT in eyes with AAU were not detected. This is in accordance with the results of Géhl et al. [2], who reported that CT values in AAU did not differ significantly from those in healthy eyes or eyes with intermediate uveitis. However, CT in the above study was measured manually in the center of all ETDRS fields, creating a CT map. In contrast, we used a novel method for the evaluation of the choroid, with calculation of CV in whole 6 mm rings around the fovea and analysis in 9 ETDRS fields. Although modern tools, such as EDI-OCT, have improved patient diagnostics and treatment monitoring, the software is still being developed. Thus, it is still recommended to check automatic measurements for accuracy. The strong point of this study is that we manually marked the scleral-uveal junction on 31 available B-scans from each examination, limiting any software mismarks. Importantly, for the first time, we performed a measurement of CV. This parameter covers more than a single measurement at a particular point or CT map based on a few marked distances, which were presented previously by several authors. The new parameters give us important information on the whole 3D volume of both Haller’s and Sattler’s choriocapillaris layers. Data were analyzed in three rings surrounding the fovea up to 6 mm and divided into 9 ETDRS fields. The analysis of CV provided more detailed information on choroidal parameters in AAU.

According to the results of the First International Workshop SUN Working Group grading both anterior chamber cells and anterior chamber flare represent standardized methods for assessing anterior inflammation [1]. However, anterior chamber flare measurement is characterized by higher interobserver agreement and narrower spread of grades in comparison with a cell count [10]. It is also easier to establish and to distinguish cases with higher inflammation degree in flare scale [10]. We detected significant positive correlations between CT changes in AAU and anterior chamber flare in most ETDRS fields. Similar observations were documented by Kim et al. [11], where a significant positive correlation between choroidal thinning after uveitis resolution and the degree of anterior chamber inflammation was detected. This observation was also confirmed by the relative choroidal vascular engorgement correlated with anterior chamber flare. Authors enrolled for the study HLA-B27 positive patients with first onset of uveitis. However, contrary to our results, the data were limited to summarized values for all ETDRS fields without subfields analysis [11].

Interestingly, choroidal parameters in AAU eyes at baseline and after treatment, as well as in fellow eyes, were negatively correlated with the age of the participants in our study. In the medical literature, age-related CT-thinning has been described previously [12–14]. In a healthy population, Wakatsuki et al. [12] presented a negative correlation between age and CT in the subfoveal quadrant, as well as the superior, inferior, nasal, and temporal quadrants, that covered a 3 mm area from the fovea. CT thinning may be the result of lipid accumulation in the macular region associated with aging. This disturbs the local paracrine passage of vascular endothelial growth factor (VEGF) from the retinal pigment epithelium to the choriocapillaris layer. VEGF is responsible for the preservation of fenestration in the choroid. The decrease in the amount of systemic water represents another factor that seems to be responsible for age-related choroidal thinning [12]. The nasal quadrant seems to be the thinnest part of the choroid [12, 14]. Sattler’s layer and the choriocapillaris layer were 200 times more predisposed to age-related changes than choroidal blood vessels with a larger diameter [12]. However, other papers presented opposite results, with a significant decrease only in Haller’s layer [14]. Importantly, the methodology used previously to establish CT was a single measurement in the subfoveal region [12, 13] or several measurements on a central scan [14].

A limitation of our study is that the analyzed group was not homogeneous in terms of the etiology of AAU and human leukocyte antigen-B27. However, AAU can be a prodromal sign of a systemic disease that may be undetectable in standard tests at the first onset of the disease. Moreover, ocular symptoms may take up to 10 years to develop after the diagnosis of a primary cause of ophthalmological symptoms.

Taken together, we conclude that evaluation of the choroid with EDI-OCT does not appear to be a reliable tool for treatment monitoring in eyes with acute anterior uveitis. On the other hand, the higher degree of the anterior chamber inflammation was associated with the bigger reduction of CT after treatment. Further investigation of the AAU population, with particular emphasis on inflammatory etiology, human leukocyte antigen-B27 status and disease activity in the anterior chamber, seems reasonable.

## Abbreviations

AAU: Acute anterior uveitis
CT: Choroidal thickness
CV: Choroidal volume
EDI-OCT: Enhanced depth imaging-optical coherence tomography
ETDRS: Early Treatment Diabetic Retinopathy Study
FE: Fellow eye
OCT: Optical coherence tomography
SD-OCT: Spectral-domain optical coherence tomography
VA: Visual acuity
VEGF: Vascular endothelial growth factor

## References

[1] Jabs DA (2005). Standardization of uveitis nomenclature for reporting clinical data. Results of the first international workshop. Am J Ophthalmol.

[2] Gehl Z, Kulcsar K, Kiss HJ, Nemeth J, Maneschg OA, Resch MD (2014). Retinal and choroidal thickness measurements using spectral domain optical coherence tomography in anterior and intermediate uveitis. BMC Ophthalmol.

[3] Yan H, Li J, Zhang J, Yang L (2017). Retinal and choroidal thickness in patients with uveitis. Ocul Immunol Inflamm.

[4] Ahn SJ, Kim JH, Lee BR (2017). Choroidal change in acute anterior uveitis associated with human leukocyte antigen-B27. PLoS One.

[5] Basarir B, Celik U, Altan C, Celik NB (2018). Choroidal thickness changes determined by EDI-OCT on acute anterior uveitis in patients with HLA-B27-positive ankylosing spondylitis. Int Ophthalmol.

[6] Gabriel M, Kruger R, Shams-Mafi F, Hermann B, Zabihian B, Schmetterer L (2017). Mapping retinal and choroidal thickness in unilateral nongranulomatous acute anterior uveitis using three-dimensional 1060-nm optical coherence tomography. Investig Ophthalmol Vis Sci.

[7] Kim M, Choi SY, Park YH (2017). Analysis of choroidal and central foveal thicknesses in acute anterior uveitis by enhanced-depth imaging optical coherence tomography. BMC Ophthalmol.

[8] Cerquaglia A, Iaccheri B, Fiore T, Lupidi M, Torroni G, Fruttini D (2016). Full-thickness choroidal thinning as a feature of Fuchs uveitis syndrome: quantitative evaluation of the choroid by enhanced depth imaging optical coherence tomography in a cohort of consecutive patients. Graefes Arch Clin Exp Ophthalmol.

[9] Kardes E, Sezgin Akçay BI, Unlu C, Ergin A (2017). Choroidal thickness in eyes with Fuchs uveitis syndrome. Ocul Immunol Inflamm.

[10] Kempen JH, Ganesh SK, Sangwan VS, Rathinam SR. Interobserver agreement in grading activity and site of inflammation in eyes of patients with uveitis. Am J Ophthalmol. 2008;146(6). 10.1016/j.ajo.2008.06.004 18687418

[11] Kim M, Kim RY, Park Y-H (2018). Choroidal vascularity index and choroidal thickness in human leukocyte antigen-B27-associated uveitis. Ocul Immunol Inflamm.

[12] Wakatsuki Y, Shinojima A, Kawamura A, Yuzawa M (2015). Correlation of aging and segmental choroidal thickness measurement using swept source optical coherence tomography in healthy eyes. PLoS One.

[13] Manjunath V, Taha M, Fujimoto JG, Duker JS (2010). Choroidal thickness in Normal eyes measured using cirrus-HD optical coherence tomography. Am J Ophthalmol.

[14] Ruiz-Medrano J, Flores-Moreno I, Peña-García P, Montero JA, García-Feijóo J, Duker JS (2017). Analysis of age-related choroidal layers thinning in healthy eyes using swept-source optical coherence tomography. Retina..

